# The Sense of Taste

**Published:** 1885-04

**Authors:** 


VOL. 32. APRIL 1885. NO. 3. 4

### THE SENSE OF TASTE.

The tongue is not the only organ used in the enjoyment of this sense, and alone it is scarcely capable of appreciating delicate flavors.

The difference between salt and sugar when placed on the tongue is hardly perceptible, provided the tongue is not allowed to touch the roof of the mouth and the lips. Indeed, the act of getting the full enjoyment of a flavor, commonly called smacking the lips, consists in bringing the tongue into contact with the roof of the mouth and the lips. By this act the substance to be tasted is spread over the surfaces of these parts, particularly of the tongue, and mixed with the saliva.

Just how this act produces taste is not exactly known ; but we do know that the tongue is covered with two layers of skin, the lower one thick and filled with nerves, and the upper one thin and porous. The nerves in the lower skin are the nerves of taste, and probably are set into vibration by the substance tasted, very much as the exquisitely sensitive nerves of the retinæ are affected by light, or the nerves of the ear by sound. At all events the sense is conveyed to the brain, where we involuntarily distinguish between pleasant and disagreeable tastes.

The nerves, moreover, of the tongue, are not all alike. In the tip of the tongue they are clustered together more closely than at the back, and transfer to the brain a different sensation. For instance, a little powdered alum placed on the back of the tongue tastes sweet, whereas on the tip it tastes acid.

The sense of taste is an almost certain guide to the wholesomeness of foods, and a monitor which warns us when we are in danger of swallowing any injurious or poisonous substance.

Poisons as a rule are extremely disagreeable to the taste, and it requires an effort to overcome the natural repugnance to them. Hence it is that accidental poisoning so rarely occurs.

In the case of foods, we soon tire of a thing as a regular diet, and the taste craves a change. Here the whole system rebels against the monotony of diet, because no one food is likely to contain all the elements of nutrition required by the body for the exercise of its functions, and soon the elements which are in excess cloy upon the taste, because the system is already supplied with them, while we crave the foods containing substances which the system lacks. A change is then demanded by nature and made manifest by the sense of taste.

If the change cannot be made, nature shows her disapproval by causing a loss of appetite, or a repugnance to the condemned article of diet.

Again, in the case of foods which are much concentrated, or have a strong flavor, like preserved fruits or syrups, the taste so on becomes dulled to the pleasure of their sweetness, because the delicate nerves which convey the impression of sweetness to the brain become fatigued, and fail to respond to the exciting cause.

There seems to be, also, a set of tastes which are in some degree complementary to each other. That is, if we taste of some intensely sweet substance, we canot detect a less degree of sweetness until the nerves have recovered from their first impression; but we can appreciate keenly any acid flavor.

To illustrate this, take a glass of lemonade, which contains substances designed to produce both sweet and acid tastes. If before drinking it we eat a lump of sugar, the lemonade will taste sour; but if we take a little clear lemon juice first, the lemonade will taste like sweetened water.

